# Antiepileptic therapy in a patient with star fruit intoxication: A case report

**DOI:** 10.1097/MD.0000000000032969

**Published:** 2023-03-03

**Authors:** Aixun Li, Baoxin Chen, Xianglan Jin, Yu Bai, Jingfeng Zhang, Chengcheng Zhang, Miaomiao Cheng, Chunyan Guo, Yu Zhang, Jing Zhou

**Affiliations:** a Graduate School, Beijing University of Chinese Medicine, Beijing, China; b Department of Neurology, Dong Fang Hospital, Beijing University of Chinese Medicine, Beijing, China; c Department of Nephrology, Dong Fang Hospital, Beijing University of Chinese Medicine, Beijing, China.

**Keywords:** antiepileptic therapy, case report, neurotoxicity, seizures, star fruit (Averrhoa carambola), uremia

## Abstract

It is rare for uremia patients to have epileptic seizures after eating star fruit, only a dozen cases are reported worldwide. Such patients usually have poor prognoses. Few patients had good prognoses, all of them were treated with expensive renal replacement therapy. At present, there is no report on the addition of drug therapy to these patients based on the initial renal replacement therapy.

**Patient concerns:**

A 67-year-old male patient with star fruit intoxication who had a history of diabetic nephropathy, hypertension, polycystic kidney, and chronic kidney disease in the uremic phase, and regular hemodialysis 3 times a week for 2 years. Initial clinical manifestations include hiccups, vomiting, speech disturbances, delayed reactions, and dizziness, which gradually progress to hearing and visual impairment, seizures, confusion, and coma.

**Diagnoses:**

This patient was diagnosed with seizures caused by star fruit intoxication. The experience of eating star fruit and the electroencephalograms can prove our diagnosis.

**Interventions:**

We performed intensive renal replacement therapy according to the recommendations in the literature. However, his symptoms did not improve significantly until he received an extra dose of levetiracetam and resumed his previous dialysis schedule.

**Outcomes:**

The patient was discharged after 21 days without neurologic sequelae. Five months after discharge, he was readmitted due to poor seizure control.

**Lessons:**

To improve the prognosis of these patients and to reduce their financial burden, the use of antiepileptic drugs should be emphasized.

## 1. Introduction

Most people know star fruit as delicious fruit. However, few of them know that one of the star fruit ingredients, caramboxin, and has neurotoxicity.^[1]^ Due to impaired excretion of certain drugs and toxins, eating star fruit can be dangerous to patients with renal dysfunction. Seizures or status epilepticus typically indicate a bad prognosis for patients with star fruit intoxication. Most physicians believe that aggressive additional renal replacement therapy (RRT) is the only way to cure patients with star fruit intoxication and that intense treatments for seizures or status epilepticus have a poor prognosis. In this particular case, the patient had multiple underlying diseases and presented with manifestations of neurological damage including seizures. After receiving medication for 21 days, he was discharged but was readmitted 5 months later with poorly controlled epilepsy.

## 2. Case presentation

**Figure 1.**
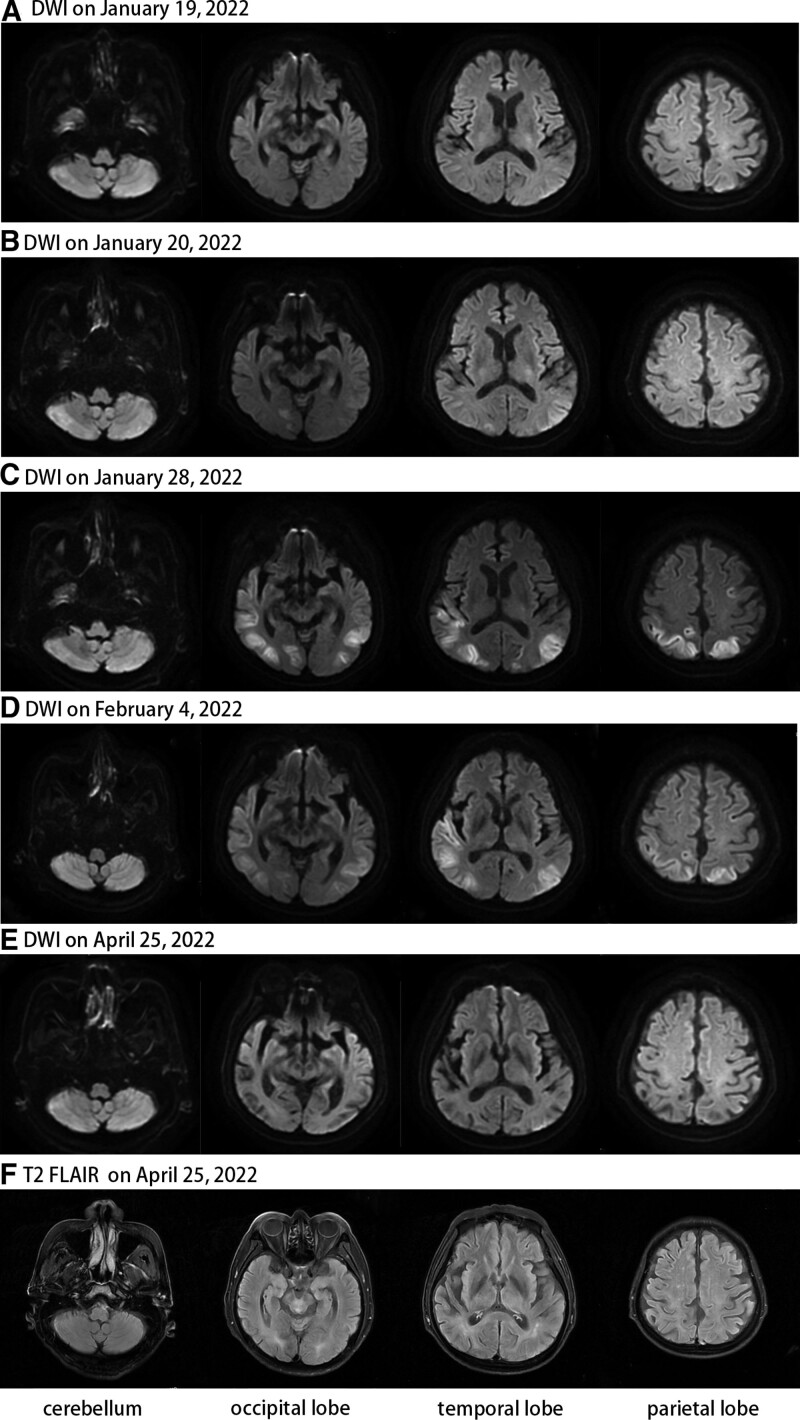
Magnetic resonance images during the first hospitalization. (A) DWI on January 19. No obvious hyperintensities were seen. (B) DWI on January 20. The bilateral cerebellar and occipital lobe have patchy slight hyperintensities. (C) DWI on January 28. The bilateral parietal lobe, occipital lobe, and temporal lobe had multiple patchy hyperintensities, which were larger than the last time, and some of them appeared new. (D) DWI on February 4. Hyperintensities are lower than last time. (E) DWI on April 25. No obvious hyperintensities. (F) T2 FLAIR on April 25. No obvious lesions left. DWI = diffusion-weighted image, FLAIR = fluid-attenuated inversion recovery.

A 67-year-old male patient with diabetic nephropathy, hypertension, and polycystic kidney disease and uremic stage of chronic renal failure (CRF) had been on regular RRT 3 times a week for 2 years. He developed symptoms such as hiccups, vomiting, speech disorders, slowed reaction, and dizziness after eating a star fruit 6 days ago. The patient was admitted via the emergency department on January 19, 2022. At the time of admission, his blood pressure was 198/76 mm Hg, pulse rate was 62/minute, respiratory rate was 16/minute and body temperature was 36.6°C. Physical examinations were not specific. His biochemical data revealed the following: creatinine levels = 866.6 μmol/L, blood urea nitrogen = 19.71 μmol/L, sodium = 140.9 mmol/L, potassium = 4.68 mmol/L, pH = 7.436, PO2 = 186 mm Hg, PCO2 = 37.4 mm Hg, HCO3 = 24.8 mmol/L, glucose = 9.58 mmol/L, hemoglobin = 126 g/L and white blood cells = 5.62 *10^9. The brain computed tomography scan (CT), and magnetic resonance images (MRI) (Fig. 1A) did not reveal any specific abnormalities. On the night of January 19, his consciousness level declined and he presented aphasia (Glasgow Coma Scale [GCS] E3V3M5). The neurologist considered acute ischemic stroke and administered thrombolysis after a CT scan to exclude cerebral hemorrhage, but there was no significant improvement in his symptoms. On the morning of January 20, he presented with a seizure of binocular upward gaze and limb twitching, which resolved with intramuscular midazolam, but still with a progressive decrease in the level of consciousness (GCS E2V2M4). It was confirmed that he had no family history of seizures or epilepsies after asking family members. The diffusion-weighted image showed slight hyperintensities in the bilateral cerebellum and occipital lobe (Fig. 1B). Physical examinations revealed bilateral pupils of equal size and roundness, pupil diameter of 3 mm, sensitive reflex to light, right-sided gaze in both eyes, normal muscle tone, bilateral Babinski (+), bilateral Chaddock (+), and the rest of the physical examinations were uncooperative. Finally, the patient was diagnosed with seizure and toxic encephalopathy caused by eating star fruits. He was admitted to the neurology department for continued treatments on January 21.

**Figure 2.**
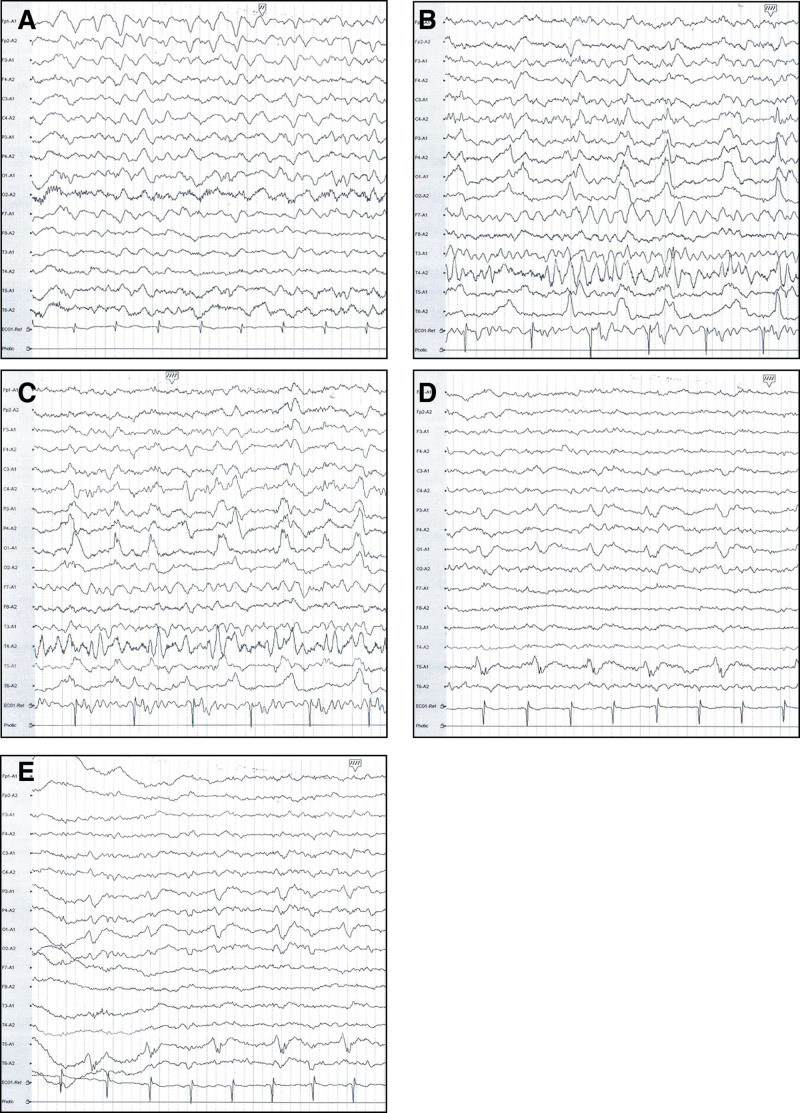
Electroencephalograms during the first hospitalization. (A) January 21. Persistent medium-high amplitude delta waves and theta waves can be seen in all montages. (B, C) January 30. Slow-waves and spike-waves periodic discharged can be seen in the bilateral parietal, occipital, and posterior temporal lobe regions. (D, E) February 4. Rhythmic delta waves and theta waves can be seen in the left parietal lobe, occipital lobe, and posterior temporal lobe. Rhythmic 1.5-2 Hz spiny slow-wave waves and multi-spike slow-waves can be seen in the left posterior temporal lobe.

The course of treatment in the neurology department is shown in Table 1. An electroencephalogram (EEG) was performed on January 21 (Fig. 2A). Our original plans were to give him RRT at the previous frequency level, 3 times a week, starting on January 21 (next on January 22), and to give levetiracetam 500mg/day followed by levetiracetam 250 mg each time after RRT to prevent future seizures. As we reviewed the literature, we found that the additional RRT is the key to improving outcomes rather than intensifying treatments of seizures or epilepsy. Therefore, we performed RRT for 6 consecutive days from January 24 to January 29 and reduced the dosage of levetiracetam to 250 mg/day. The brain MRI on January 28 (Fig. 1C) showed that the hyperintensities were larger than the last time, and some appeared new.

**Table 1:** The course of treatment during neurology department.

Date	The patient condition	Auxiliary examination	Interventions
Jan 21st to Jan 23^rd^	Occasional hiccups, no nausea and vomiting, anuria, fecal incontinence, hearing and vision impairments, coma, right-sided gaze in both eyes and no recurrence of seizures.	•The EEG on Jan. 21st. (Fig. 2A).	•RRT for 3 times a w. (next on Jan. 22nd) •Levetiracetam 500mg/d followed by 250 mg each time after RRT.
Jan 24th to Jan 30^th^	Hearing and vision impairments, confusion, agitation and occasional right-sided gaze.	•The brain MRI on Jan. 28 (Fig.1C). •The EEG on Jan. 30th (Fig.2B and C).	•RRT for 6 consecutive d. •Levetiracetam 250 mg/d
Jan 31st to Feb 10^th^	The condition improved rapidly.	•The brain MRI on Feb. 4th (Fig. 1D) •The EEG on Feb. 8th (Fig.2D and E).	•RRT for 3 times a week. •Levetiracetam 500mg/d followed by 250 mg each time after RRT.
Feb 11^th^	Discharged with complete resolution of symptoms and no neurological sequelae.		•RRT for 3 times a week at the out-patient hemodialysis unit. •Levetiracetam 500mg/d and followed by 250 mg each time after RRT.

EEG = electroencephalograms, MRI = magnetic resonance images, RRT = renal replacement therapy.

On January 30, after intensive RRT for 6 consecutive days, he still had occasional right-sided gaze, hearing and vision impairments, confusion, and agitation (GCS E2V3M4). The EEG (Fig. 2B and C) on that day confirmed that his condition had not improved significantly. On January 31, we tentatively increased the dosage of levetiracetam again to 500mg/day, restored the frequency of RRT to what he received before (3 times a week), and gave levetiracetam 250 mg after every time of RRT. The patient condition improved rapidly after the above treatments. Four days later, on February 3, he regained consciousness (GCS E4V5M6) and could recall the onset of the disease. He was also able to walk and to complete simple activities of daily life independently. The brain MRI on February 4 (Fig. 1D) and the EEG on February 8 (Fig. 2D and E) both indicated that his condition was better than before.

The patient was discharged on February 11, 2022, 21 days after admission, with complete resolution of symptoms and no neurological sequelae, but physicians still recommended him taking levetiracetam for at least 6 months. At the same time, he continued to receive regular hemodialyses at the out-patient hemodialysis unit after discharge. The brain MRI on April 25, 2022 was unremarkable and had no residual lesions (Fig. 1E and F).

**Figure 3.**
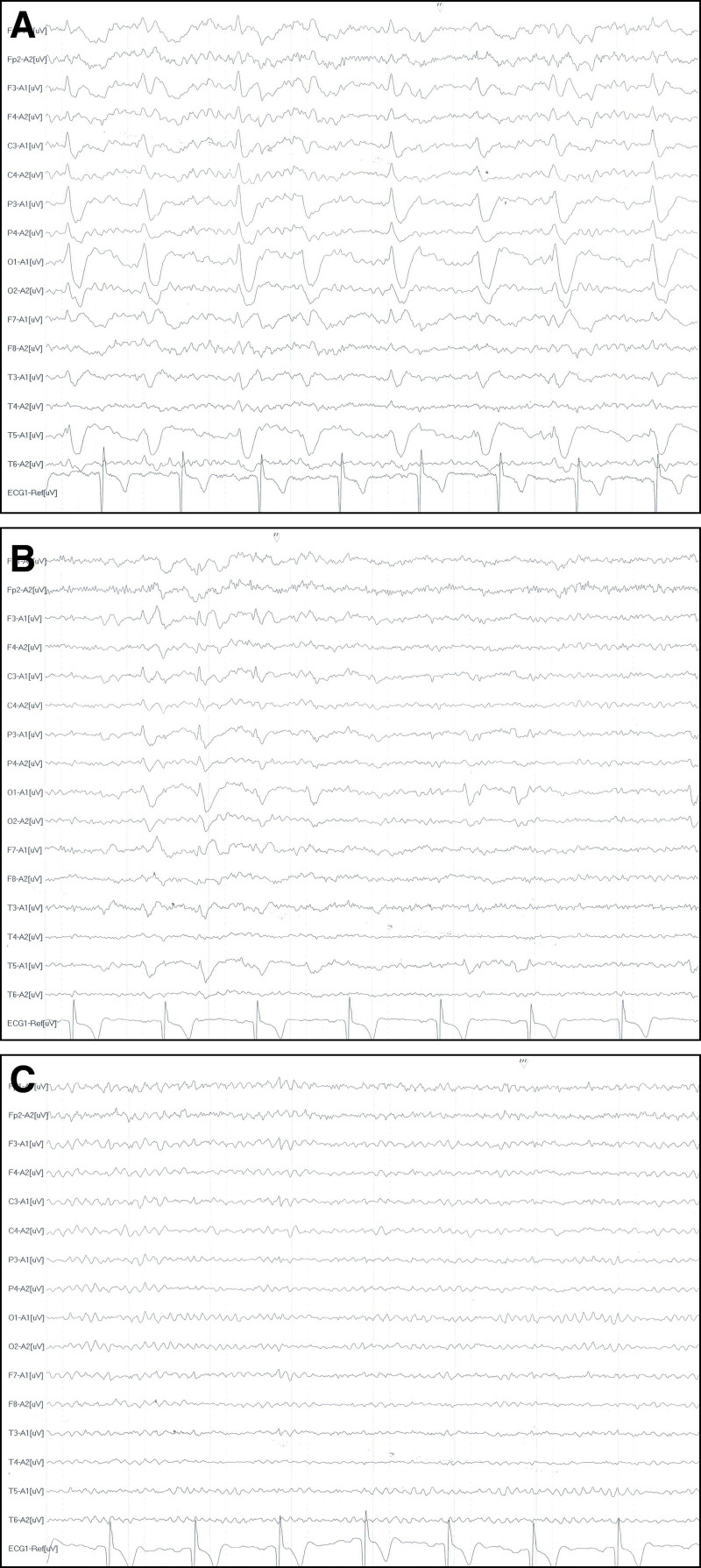
Electroencephalograms during the second hospitalization. (A) July 8. Slow-wave and sharp-wave periodic emission. (B) July 11. Scattered or rhythmic medium-high amplitude sharp waves. (C) July 20. Most of the abnormal waves disappear.

He was readmitted to the hospital again on July 8, 2022, with symptoms of confused speech and abnormal behavior. On MRI and CT, no appreciable abnormalities were discovered. A large number of spike-waves and slow-waves periodic discharged on the EEG on the day of admission (Fig. 3A). We consequently assumed that his poorly controlled epilepsy was to blame for his symptoms this time. In terms of therapy, we just upped the levetiracetam dosage to 500 mg Bid while maintaining the frequency of his RRT. The second dose was given after the RRT on the day of the RRT. Epileptiform discharges gradually disappeared on the EEG on July 11 and July 20 (Fig. 3B and C). This patient was discharged on the 12th day after the second admission, also with no neurological sequelae. He said that he did not feel any discomfort after being discharged from the hospital.

## 3. Discussion

Star fruit is a tropical fruit popular in many countries and regions. However, only some people are familiar with its nephrotoxic and neurotoxic effect. Oxalate causes nephrotoxicity, which is most common in patients with normal renal function.^[2–4]^ Acute tubular necrosis and interstitial nephritis can be caused by oxalic acid crystals deposited in the renal tubules.^[5]^ Caramboxin is the cause of neurotoxicity, which is primarily seen in patients with abnormal renal function.^[1]^ It is impossible to estimate the incidence of neurotoxicity in individuals with normal renal function because so few of them are reported in the previous literature.

Martin was the first to report star fruit intoxication in patients on regular dialysis.^[6]^ Eight patients developed persistent hiccups after ingesting star fruit, but no other symptoms were described. Since then, there have been more reports of hiccups, vomiting, and even seizures and comas after ingesting star fruit, but the root cause of these symptoms was unclear. Until 2013, Garcia-Cairasco extracted this neurotoxic substance from star fruit, named it caramboxin, and demonstrated its neuroexcitatory properties.^[1]^ Caramboxin has decreased clearance in patients with abnormal renal function and produces excitatory effects on the central nervous system. Neto classified the neurotoxic effects of star fruit into 3 levels of intoxication: mild, moderate and severe intoxication.^[7]^ Coma, seizures or status epilepticus and shock are signs of severe intoxication. This result suggests that seizure is a poor prognostic factor for star fruit intoxication and deserves the attention of physicians. In the case we report here, the symptoms from initial hiccups and vomiting to impaired consciousness were largely consistent with those described in previous reports. This patient also presented seizures and coma, which were classified as severe intoxication according to Neto classification. It may suggest a poor prognosis.

The differences between the case we reported this time and the previous ones are the following.

First, in terms of treatment. In previously published cases of this disease, the choice of renal replacement therapy modality is the focus of treatment for most physicians. This is the first report of a treatment focus on antiepileptic therapy. As recommended in previous literature, we performed RRT for 6 consecutive days while administering 250 mg of levetiracetam daily. However, the outcomes after 6 days were unsatisfactory. There was no significant improvement in the patient symptoms, which MRI and EEG confirmed. Then, we considered whether the consecutive RRT resulted in an excessive clearance of levetiracetam and an insufficient blood level to control epilepsy. Because of this, we experimented with decreasing the frequency of RRT and increasing the levetiracetam dosage. His condition significantly improved after 4 days. The optimal treatment for patients with star fruit intoxication has been controversial. In the Neto classification, it was proved that aggressive RRT is effective for patients with mild and moderate intoxication. In contrast, there are still significant differences in outcomes for patients with severe intoxication, even they received timely supportive therapy and aggressive RRT. Despite this, it is undeniable that aggressive RRT is currently the only way to improve patient prognosis. This is why we decided to increase the frequency of RRT in the first place. However, the subsequent change in this patient condition differed from what had previously been reported in the literature.

Second, on the prognosis. The patient we reported had several diseases and was in the uremic phase of CRF. Furthermore, he had seizures and was severe intoxication according to Neto grading criteria, with a likely poor prognosis. The short-term prognosis of the patient we report is favorable. But the star fruit intoxication seems to lower the threshold of seizures from our follow-up results. The outcomes of patients with star fruit intoxication vary widely. Some patients recovered in a short time without any sequela and did not need other therapy, some patients recovered but still needed to continue dialysis or take antiepileptic drugs, and others eventually died. According to previous reports, 26% of patients with star fruit intoxication had seizures. The mortality rate of these patients who developed seizures was 61%, which was significantly higher than those without seizures.^[8]^ The reasons for these individual differences are still controversial. What is certain is that there is no association between the type of star fruit and the form in which it was ingested and the severity of intoxication symptoms or mortality.^[7,9]^ A paper reviewed the characteristics of patients with star fruit intoxication in the last decade: 63.2% of patients were male and 69.1% of patients had previous renal abnormalities (63.8% of these patients were on hemodialysis or peritoneal dialysis). Meanwhile, patients with abnormal kidney function had a worse prognosis compared with healthy individuals.^[10]^ However, it was not verified that the degree of decline in the renal function directly predicted the severity of caramboxin-induced neurological impairment.

Lastly, there is no published information about the follow-up of individuals who have previously suffered from star fruit intoxication. For this patient, we followed up for 6 months. In the fifth month after discharge, and he was readmitted for suspected poorly controlled epilepsy. It cannot be excluded that a history of star fruit intoxication decreased the threshold for seizures.

These are some of the ideas that have emerged through caring for this patient. We cannot explain why the patient symptoms did not improve significantly after consecutive RRT and why the seizures were induced during the second hospitalization because there is no way to detect the amount of caramboxin in the body or measure the blood concentration of levetiracetam on time. Most physicians focus on the choice of RRT when caring for these kinds of patients. We think using antiepileptic drugs is just as important as choosing the right RRT for people with seizures or persistent status epilepticus. At the same time, the relationship between the dose of antiepileptic drugs and the choice of RRT needs to be explored. Clinicians who see a patient with CRF and feel they may be experiencing symptoms should also consider whether the patient has ever consumed star fruit because some of the symptoms are similar to those of metabolic encephalopathy and stroke.

## Author contributions

**Methodology:** Yu Bai, Jingfeng Zhang, Chengcheng Zhang, Miaomiao Cheng.

**Writing – original draft:** Aixun Li, Chunyan Guo, Yu Zhang.

**Writing – review & editing:** Baoxin Chen, Xianglan Jin, Jing Zhou.
